# Over-Expression and Prognostic Significance of FN1, Correlating With Immune Infiltrates in Thyroid Cancer

**DOI:** 10.3389/fmed.2021.812278

**Published:** 2022-01-24

**Authors:** Qi-Shun Geng, Tao Huang, Li-Feng Li, Zhi-Bo Shen, Wen-Hua Xue, Jie Zhao

**Affiliations:** ^1^Department of Pharmacy, The First Affiliated Hospital of Zhengzhou University, Zhengzhou, China; ^2^Huanghe Science and Technology University, Zhengzhou, China; ^3^Internet Medical and System Applications of National Engineering Laboratory, The First Affiliated Hospital of Zhengzhou University, Zhengzhou, China

**Keywords:** thyroid cancer, biomarker, FN1, immunity, survival

## Abstract

**Background:**

Thyroid cancer (THCA) is a malignancy affecting the endocrine system, which currently has no effective treatment due to a limited number of suitable drugs and prognostic markers.

**Methods:**

Three Gene Expression Omnibus (GEO) datasets were selected to identify differentially expressed genes (DEGs) between THCA and normal thyroid samples using GEO2R tools of National Center for Biotechnology Information. We identified hub gene *FN1* using functional enrichment and protein-protein interaction network analyses. Subsequently, we evaluated the importance of gene expression on clinical prognosis using The Cancer Genome Atlas (TCGA) database and GEO datasets. MEXPRESS was used to investigate the correlation between gene expression and DNA methylation; the correlations between *FN1* and cancer immune infiltrates were investigated using CIBERSORT. In addition, we assessed the effect of silencing *FN1* expression, using an *in vitro* cellular model of THCA. Immunohistochemical(IHC) was used to elevate the correlation between *CD276* and *FN1*.

**Results:**

*FN1* expression was highly correlated with progression-free survival and moderately to strongly correlated with the infiltration levels of M2 macrophages and resting memory CD4+ T cells, as well as with *CD276* expression. We suggest promoter hypermethylation as the mechanism underlying the observed changes in *FN1* expression, as 20 CpG sites in 507 THCA cases in TCGA database showed a negative correlation with *FN1* expression. In addition, silencing *FN1* expression suppressed clonogenicity, motility, invasiveness, and the expression of CD276 *in vitro*. The correlation between FN1 and CD276 was further confirmed by immunohistochemical.

**Conclusion:**

Our findings show that *FN1* expression levels correlate with prognosis and immune infiltration levels in THCA, suggesting that *FN1* expression be used as an immunity-related biomarker and therapeutic target in THCA.

## Introduction

Thyroid carcinoma (THCA) is the most common type of endocrine cancer (1). Its prevalence has sharply increased in recent decades. In the US, the annual incidence of thyroid cancer increased from 4.9/100,000 in 1975 to 14.3/100,000 in 2009 (2). The observed increase in the incidence is partly due to the increased detection rate. In Beijing, China, the detection rate significantly increased from 16.8% in 1994 to 69.8% in 2015 (*P* < 0.01) (3). THCA can be subclassified into several histological subtypes, which include papillary thyroid carcinoma (PTC), follicular thyroid carcinoma (FTC), undifferentiated or anaplastic thyroid carcinoma (ATC), and medullary thyroid carcinoma (MTC). THCA occurs mostly in young adults (the average age of diagnosis is 40 years), and more frequently in females (4). The most common treatment of thyroid cancer consists of surgical resection combined with radiotherapy or chemotherapy. However, for PTC, ATC, and MTC, a satisfactory resection is not always feasible, and even after radiotherapy, the risk of cancer recurrence is still high (5, 6). In addition, recently personalized therapy approaches directed against specific targets have become available, only a few suitable targets have been identified thus far. Although the survival rate of patients with thyroid cancer is very high, with the rapid increase in THCA incidence, this disease poses a serious threat to human health (7).

Immune-related processes play an important role in the development of thyroid cancer, and hence, immunotherapy strategies are considered the most promising candidates for the treatment of thyroid cancer in the future (8). Many studies have shown that tumor-infiltrating lymphocytes, such as tumor-associated macrophages, tumor-associated dendritic cells, and tumor-infiltrating neutrophils, affect the prognosis of THCA patients and the efficacy of chemotherapy and immunotherapy (9). Therefore, there is an urgent need to understand which immune cells play a role in the development of THCA, as well as to explore novel immune-related biomarkers that could aid in the diagnosis and prognosis of this disease.

Fibronectin 1 (*FN1*) encodes a glycoprotein present in a soluble dimeric form in the plasma and a dimeric or multimeric form at the cell surface and in the extracellular matrix. FN1 is involved in cell adhesion and migration processes during embryogenesis, wound healing, blood coagulation, host defense, and metastasis, as well as in cell proliferation (10). Multiple studies have established its involvement in the development of cancer, including oral squamous cell carcinoma (11), renal cancer (12), and thyroid cancer (13). Previous studies have indicated that FN1 is involved in NKp46 receptor-mediated interferon-γ (IFN-γ) production by natural killer cells, with respect to the control of tumor architecture and metastasis (14). BAG5 also promotes invasion of papillary thyroid cancer cells *via* upregulation of FN1 at the translational level (15). In addition, FN1 plays an important role in glioblastomagrowth and invasion, and is correlated with the low elasticity of thyroid nodules and Malignancy of THCA (16, 17). These findings suggest that FN1 has multifaceted functional roles in tumor progression.

In this study, we comprehensively analyzed the correlation between *FN1* expression with the prognosis of patients with THCA, as well as with the presence of tumor-infiltrating immune cells. Our findings highlight the important role of *FN1* expression in THCA, and suggest a potential correlation between *FN1* expression and tumor-immune interactions. Furthermore, the results were validated by immunohistochemistry and cell biology experiments, which indicate that *FN1* is a potential prognostic and immunity-related biomarker in THCA and could potentially be used as a drug target in THCA therapy.

## Materials and Methods

### Cell Lines and Reagents

The cell lines B-CPAP and KTC-1, belonging to the thyroid cancer cell lines, were obtained from the Institute of Biochemistry and Cell Biology of the Chinese Academy of Sciences, Shanghai, China. All cells were cultured in Roswell Park Memorial Institute (RPMI) 1640 medium (Gibco, Grand Island, NY, USA) supplemented with 10% fetal bovine serum (10%FBS), 100 U/mL penicillin, and 100 mg/mL streptomycin (Invitrogen, Carlsbad, CA, USA). Cells were incubated in a 5% CO_2_/95% O_2_ in a humidified atmosphere at 37°C.

### Data Sources and Identification of Differentially Expressed Genes (DEGs)

TCGA is a landmark cancer genomics program that characterized over 20,000 primary cancer samples, spanning 33 cancer types and matched to the respective normal samples. Samples are molecularly characterized, and multi-omics data are provided, including gene transcripts, miRNA expression data, and DNA methylation state data. Additionally, it contains abundant and standardized clinical data. All datasets used were downloaded from the Cancer Genomics Browser website of the University of California, Santa Cruz (18). The GEO database (http://www.ncbi.nlm.nih.gov/geo/) stores original submitter-supplied records (series, samples, and platforms), as well as the curated datasets. Using the selection criteria of a number of samples >20 and < 1,000, we selected three gene expression profiles [GSE33630 (19), GSE58545 (20), and GSE60542 (21)]. Among them, GSE33630 (105 samples) and GSE60542 (45 samples) are based on the GPL570 platform, and GSE58545 (92 samples) was obtained with the GPL96 platform. According to the commonly used threshold parameters (adjusted *P* < 0.05, |log_2_FoldChange| ≥ 2.0) (22), we determined the DEGs between THCA and normal thyroid samples using the GEO2R online analysis tool, accessible *via* the National Center for Biotechnology Information website. Then, the interactive tool Venny2.1.0 (23) was used to create a Venn diagram of the DEGs to determine the common DEGs between the three analyzed gene expression profiles.

### Functional Enrichment Analysis and Identification of Hub Genes

Gene Ontology (GO) is an initiative that provides a standardized classification of genes by accounting for their functions, biological pathways they participate in, and the cell localization of the corresponding proteins. Genes are categorized into three domains: biological process (BP), molecular function (MF), and cellular component (CC) (24). We annotated the identified DEGs according to the GO classification system using the Database for Annotation, Visualization, and Integrated Discovery (DAVID) tool (https://david.ncifcrf.gov/) (25). The threshold criteria to determine the significantly enriched GO terms were *P* < 0.05 and gene counts ≥ 5. To identify the hub genes, a protein-protein interaction (PPI) network was constructed for the DEGs that had been annotated in the category BP (*P* < 0.05) using Search Tool for the Retrieval of Interacting Genes (STRING) (http://string-db.org/) (26). PPI pairs with a combined confidence score ≥ 0.4, were visualized using Cytoscape (version 3.7.2) (27). The Cytoscape plugin Molecular Complex Detection (MCODE) (version 1.4.2), an app to cluster any given network, was used to identify the most important module in the PPI network, and the plugin CytoHubba was used to identify the hub genes in the PPI networks by calculating the degree of connectivity between DEGs. The selection criteria were as follows: MCODE score > 5 points, degree cut-off = 2, node score cut-off = 0.2, maximum depth = 100, and k-score = 2.

### Correlation Between Gene Expression and Survival

ONCOMINE is an online cancer microarray database (www.oncomine.org) (28). Gene expression profiles from the website were used to analyze the transcription levels of *FN1* in THCA. Furthermore, the correlation between *FN1* expression and progression-free survival (PFS) and clinical parameters was analyzed using the TCGA database. In addition, the UALCAN (29), a web resource to analyze cancer OMICS data, was used to investigate the correlation between *FN1* expression and cancer stage, and between *FN1* expression and promoter methylation level.

### Methylation and Immunity Correlation Analysis

MEXPRESS (https://mexpress.be/) is a data visualization tool designed for easy visualization of TCGA expression, DNA methylation, and clinical data, as well as the correlations between them (30, 31). We used this tool to investigate the correlation between hub gene expression and the degree of methylation of the gene promoters. CIBERSORT is an analytical tool used to estimate the abundance of cell types in a mixed cell population using gene expression data (32). We used this tool to assess the degree of immune infiltration. The co-expression analysis of *FN1* and B7 family members (including *CD274, CD80, CD86, CD276, CD273, CD275, B7-H4, B7-H5, CD28, B7-H7, CD152, CD279, CD278, TLT-2*, and *NKp30*) was assessed in the normal thyroid samples and in the THCA samples from TCGA database. The correlation between *FN1, CD273, CD274, CD275, B7-H4*, and *CD276* was further analyzed in the THCA cohort. GEPIA (http://gepia.cancer-pku.cn/detail.php) (33) and TIMER (39) were used to plot the expression scatterplots between any pair of genes in a given cancer type, while including the Spearman correlation and the statistical significance.

### Silencing of FN1 by Small Interfering RNA (siRNA)

The siRNA targeting human *FN1* (siFN1) and a non-specific scrambled siRNA sequence (siNC) were purchased from Shanghai Gene Pharma and transiently transfected into B-CPAP and KTC-1 cells using Lipofectamine 3000 (Invitrogen, Carlsbad, CA, USA) according to the manufacturer's instructions. The target sequence in *FN1* was: 5′-CAGUCAAAGCAAGCCCGGUUGUUAU-3′. Subsequently, assays were performed 48 h after the transfection. Cell viability was assessed 24, 48, or 72 h after transfection using the commercial kit Cell Counting Kit-8 (CCK-8, Beyotime Biotechnology, China) according to manufacturer's instructions.

### Quantitative Reverse Transcription-Polymerase Chain Reaction (RT-qPCR)

RNA was isolated using TRIzol reagent (Invitrogen, Carlsbad, CA, USA), followed by transfer into RNA-free EP tubes and storage at −80°C. Complementary DNA (cDNA) was synthesized from total RNA using the PrimeScript RT reagent Kit (Takara, Dalian, China), and PCR was performed using the SYBR Green RT-PCR Kit (AG11701, Accurate Biotechnology, Hunan, Co., Ltd). PCR was performed on the StepOne Plus Real-Time PCR System (Applied Biosystems, Foster City, CA, USA). Data were analyzed using the 2^−ΔΔCT^ method. The primer sequences used in the experiment are as follows: FN1, 5′-CGGTGGCTGTCAGTCAAAG-3′ (forward), 5′-AAACCTCGGCTTCCTCCATAA-3′ (reverse); CD276, 5′-CTCCCTACAGCTCCTACCCTC-3′ (forward), 5′-TGGTCTGTGTATCGCATCCTT-3′ (reverse).

### Immunohistochemistry (IHC) Staining

Samples from cancer patients were obtained from thyroid cancer arrays (DC-Thy11004 Avira Biotechnology Co., Ltd., China), which included 24 cases of thyroid cancer and corresponding paracancerous tissues. Tumor tissues chip were deparaffinized and rehydrated, followed by antigen retrieval. The sections were then blocked with 5% BSA in PBS and incubated with FN1 antibody (1 mg/mL, 1:200; Affinity) and CD276 antibody (1 mg/mL, 1:200; Affinity) at 4°C overnight. After three times washing, tissue sections were incubated with the secondary antibody conjugated with streptavidin–horseradish peroxidases for 1 h at room temperature. The slides were stained with 3, 3-diaminobenzidine tetrahydrochloride (DAB), and the nuclei were counterstained with hematoxylin. Marker density was scored independently by two investigators as follows: 0, negative; 1, weak; 2, moderate; or 3, strong.

### Statistical Analyses

All statistical analyses were performed using GraphPad Prism 5.0 (San Diego, CA, USA) and R version 3.6.1. Data from independent experiments performed in triplicate are presented as mean ± standard deviation (SD). Multivariate survival analysis was carried out for all parameters that were significant in the univariate analysis using the Cox regression model. To analyze the significance of differences between groups, unpaired two-tailed Student's *t*-test and one-way analysis of variance (ANOVA) were performed, and multiple comparisons were accounted for using Bonferroni's correction. Differences were considered significant at *P* < 0.05.

## Results

### Identification of the Common DEGs Between the Datasets Used

**Figure 1:**
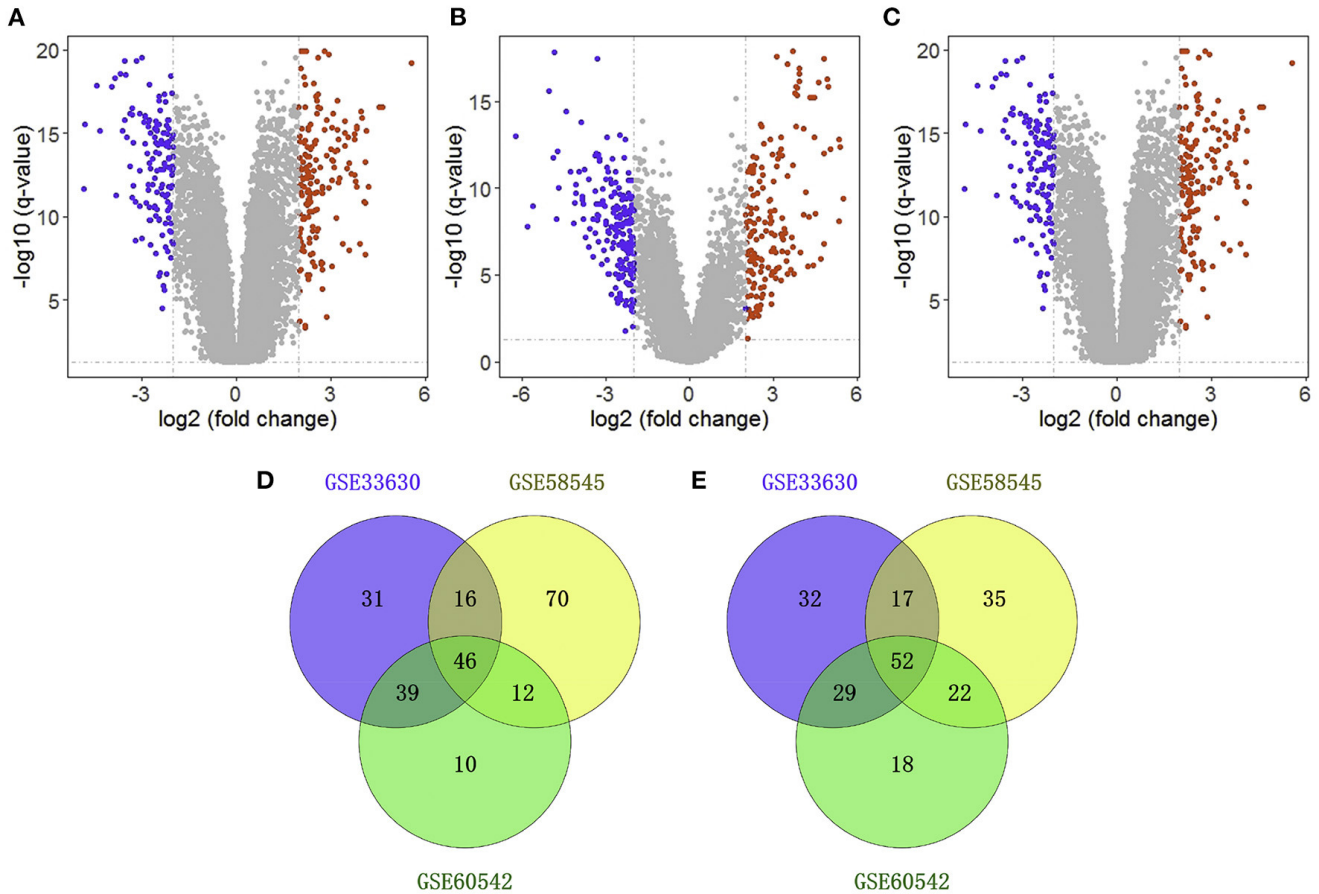
Identification of differentially expressed genes (DEGs). Volcano plot showing the differentially expressed genes of GSE33630 **(A)**, GSE58545 **(B)**, and GSE60542 **(C)**. Veen diagram of downregulated **(D)** and upregulated **(E)** common DEGs to three gene expression profiles.

We obtained three gene expression profiles (GSE33630, GSE58545, and GSE60542) from the GEO database. GSE33630 includes 45 normal thyroid samples and 60 THCA samples; GSE58545 includes 18 normal thyroid samples and 27 THCA samples; GSE60542 includes 30 normal thyroid samples and 33 THCA samples. According to the conventional criteria (adjusted *P* < 0.05 and |log_2_FoldChange| ≥ 2.0), 263 genes were identified as DEGs in GSE33630, of which 133 were downregulated and 130 were upregulated; moreover, GSE58545 included 270 DEGs, including 144 downregulated genes and 126 upregulated genes, and GSE60542 contained 228 DEGs, including 107 downregulated genes and 121 upregulated genes (Figures 1A–C). A Venn diagram showed that 98 genes were differentially expressed in the three datasets, of which 46 were downregulated, and 52 were upregulated (Figures 1D,E).

### Functional Enrichment Analysis of the Common DEGs

In this study, we performed GO functional analysis of the common DEGs using the tool DAVID. Then, we filtered the results to improve the confidence according to the standard criteria (*P* < 0.05 and gene counts ≥ 5) (Supplementary Table 1). GO analysis showed that the common DEGs were mainly enriched in the CC category, including the plasma membrane, extracellular exosome, extracellular region, and extracellular space. DEGs annotated as BP were enriched in cell adhesion, signal transduction, extracellular matrix organization, positive regulation of gene expression, positive regulation of cell proliferation, blood coagulation, wound healing, platelet degranulation, and nervous system development, all of which are processes associated with the occurrence and development of tumors.

### Identification and Analysis of the Hub Genes

**Figure 2:**
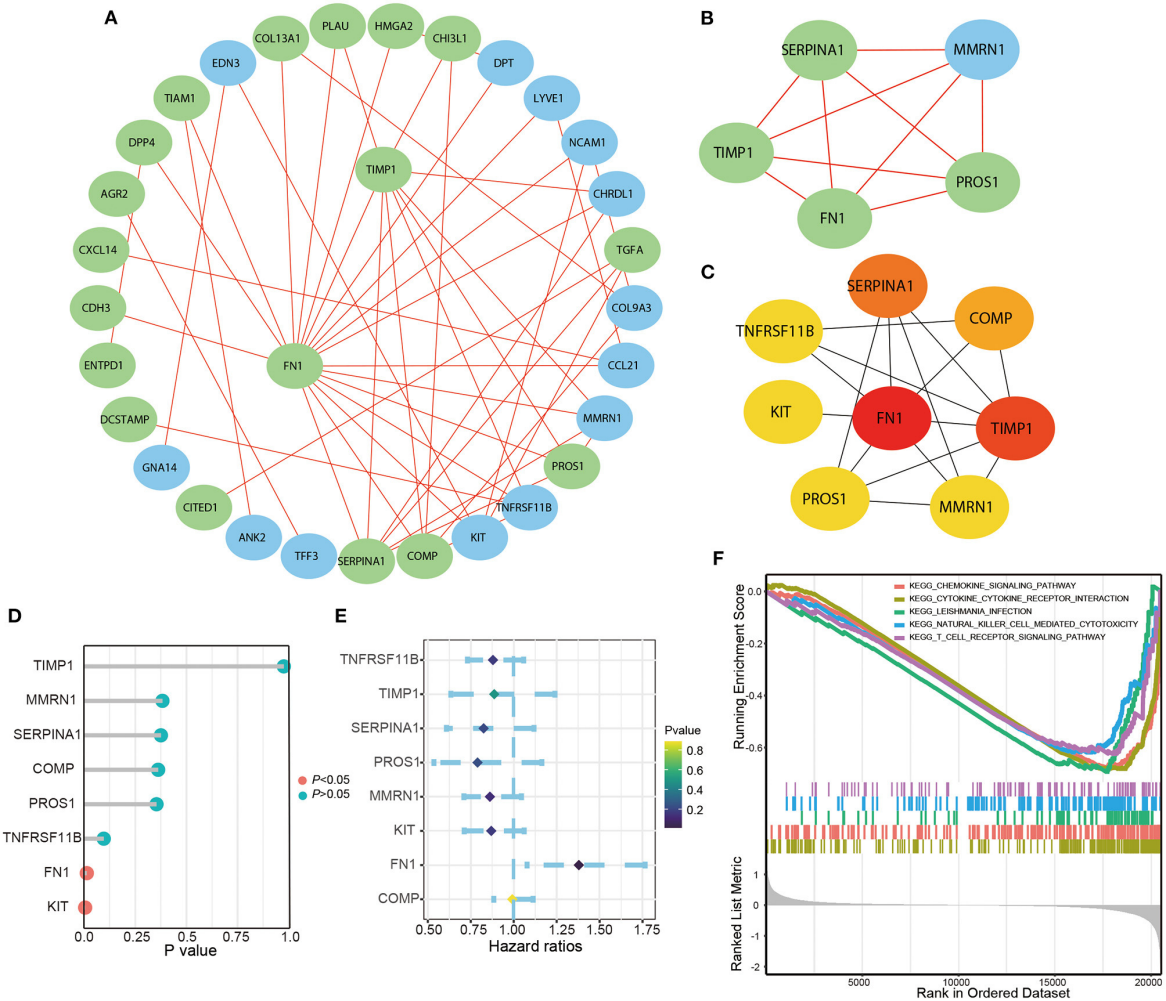
The Screening and identification of key genes. **(A)** PPI network constructed with the DEGs in biological progress. **(B)** The most significant module obtained from PPI network. **(C)** The top eight hub genes. The logrank analysis of hub genes **(D)** and the multivariate cox logistic regression analysis **(E)** of hub genes based on the TCGA database. **(F)** The significant kegg pathways of FN1 in THCA obtained by GSEA.

We selected 46 DEGs involved in specific BP (*P* < 0.05) to build the PPI network (Figure 2A; Table 1). The most important module was obtained using the plugin MCODE in Cytoscape (Figure 2B). The top eight genes, including *FN1, TIMP1, SERPINA1, COMP, PROS1, MMRN1, KIT*, and *TNFRSF11B*, were identified as potential hub genes according to the degree score generated by the plugin CytoHubba (Figure 2C; Table 2). This was consistent with their enrichment in the top module determined using MCODE. Among them, *FN1* had the highest degree of connectivity in the PPI network. Furthermore, logrank regression and multivariate Cox regression analysis were used to calculate the correlation between gene expression and PFS (Figures 2D,E), indicating *FN1* appeared to be the most attractive drug target and prognostic marker. GSEA was used to perform kegg analysis for FN1. The results suggested that most of the involved significant pathways including chemokine signaling pathway, cytokine receptor interaction, Leishmania infection, natural killer cell-mediated cytotoxicity, and T cell receptor signaling pathway, as it has been established that FN1 plays a crucial role in tumor architecture and controls metastasis (Figure 2F) (13).

**Table 1:** The information related to biological processes of statistical significance.

**Term**	**Describe**	***P*-value**	**Gene**	**Count**
GO:0007155	Cell adhesion	1.61E-06	PLXNC1, CYP1B1, CLDN10, MMRN1, CDH3, NCAM1, LAMB3, LYVE1, CDH16, SORBS2, COMP, ENTPD1, DPT, FN1	14
GO:0007165	Signal transduction	0.012464	GNA14, EDN3, CRABP1, RAP1GAP, KIT, HMGA2, LYVE1, TNFRSF11B, CXCL14, ANK2, TENM1, IGSF1, GDF15, PLAU	14
GO:0030198	Extracellular matrix organization	0.000814	CSGALNACT1, TNFRSF11B, LAMB3, COL9A3, COL13A1, COMP, FN1	7
GO:0010628	Positive regulation of gene expression	0.00353	ANK2, KIT, HMGA2, CDH3, AGR2, CITED1, FN1	7
GO:0008284	Positive regulation of cell proliferation	0.046698	EDN3, TIAM1, TGFA, KIT, DPP4, FN1, TIMP1	7
GO:0007596	Blood coagulation	0.046698	SERPINA1, MMRN1, ENTPD1, PROS1, PAPSS2, PLAU	6
GO:0042060	Wound healing	0.046698	TGFA, TFF3, CDH3, FN1, TIMP1	5
GO:0002576	Platelet degranulation	0.046698	SERPINA1, MMRN1, PROS1, FN1, TIMP1	5
GO:0007399	Nervous system development	0.046698	CSGALNACT1, CHRDL1, TENM1, BEX1, MPPED2	5

**Table 2:** Hub genes with higher degree of connectivity.

**Gene symbol**	**Gene title**	**Degree**	**Gene nature**
FN1	Fibronectin 1	17	UP
TIMP1	TIMP metallopeptidase inhibitor 1	9	UP
SERPINA1	Serpin family A member 1	6	UP
COMP	Cartilage oligomeric matrix protein	5	UP
PROS1	Protein S (alpha)	4	UP
MMRN1	Multimerin 1	4	DOWN
KIT	KIT proto-oncogene receptor tyrosine kinase	4	DOWN
TNFRSF11B	TNF receptor superfamily member 11b	4	DOWN

### Expression Levels of *FN1* in THCA and Evaluation of Its Value as a Prognostic Marker

**Figure 3:**
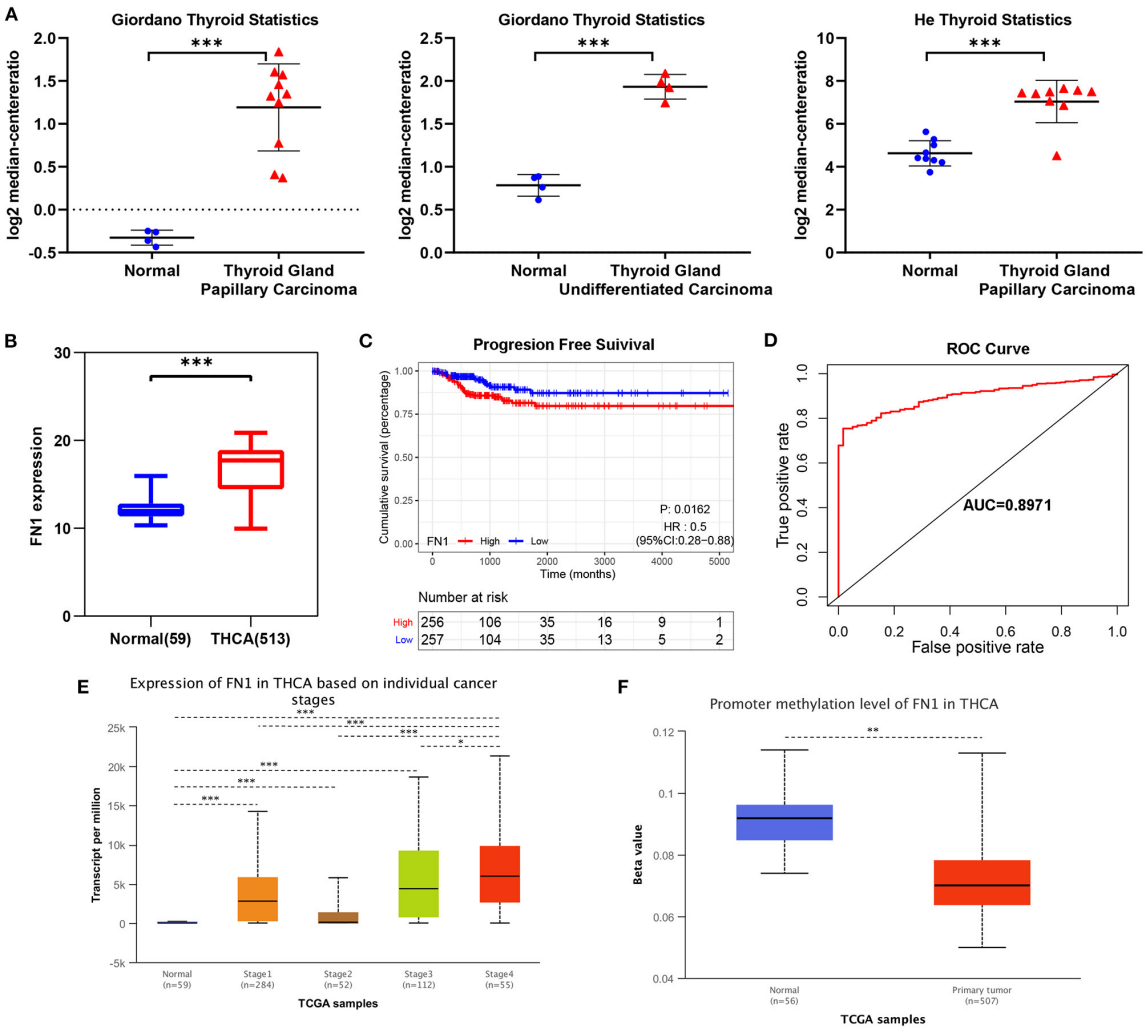
Transcriptional expressions of FN1 significantly correlated with poor survival outcomes in THCA patients from TCGA cohort. **(A)** The expression of FN1 in different subtypes of THCA. **(B)** The expressions of FN1 in THCA patients from TCGA-THCA cohort, **(C)** The correlation between the expression of FN1 and PFS, **(D)** ROC curve with an AUC of 0.8971. Transcriptional expression of FN1 was significantly correlated with individual cancer stages **(E)** and promoter methylation **(F)**. *p < 0.05, **p < 0.01, ***p < 0.001.

We analyzed the expression levels of *FN1* in THCA using the Oncomine database. We found that *FN1* expression was upregulated in almost all different subtypes of THCA, including PTC and ATC (Figure 3A). We validated these results using the TCGA database, in which *FN1* was also significantly high expressed in THCA samples than in normal thyroid samples (*P* < 0.05) (Figure 3B);. and *FN1* expression level was also correlated with PFS of THCA (*P* < 0.05) (Figure 3C). The area under the curve (AUC) of *FN1* from the TCGA dataset was 0.8971 (Figure 3D), highlighting the value of *FN1* as a diagnostic marker in THCA. We compared the clinical characteristics (including age, sex, location, clinical stage, progression state, lymph node metastasis, and distant metastasis) between the *FN1*-high expression and *FN1*-low expression groups, and observed statistically significant differences in lymph node metastasis, distant metastasis, and progression state, although no significant differences were identified for other clinical features (Table 3). Furthermore, univariate and multivariate Cox regression models revealed that clinical stage and *FN1* expression were independent prognostic factors for PFS in patients with THCA (Table 4). In addition, analysis of the UALCAN database showed that *FN1* expression levels were closely correlated to cancer stage (Figure 3E) and that the degree of methylation of the *FN1* promoter was lower in THCA than that in normal tissues (Figure 3F), indicating that *FN1* may be involved in the development of THCA.

**Table 3:** Comparison of clinical characteristics between low FN1 group and high FN1 group in THCA cohort.

**Variable**	**Case no. (%)**	**FN1**		***P***
		**High**	**Low**	
Sample	505	253	252	
Age (Year)				
≥60	118	62	56	0.544
<60	387	191	196	
Gender				
Female	366	182	184	0.786
Male	139	71	68	
Clinical stage				
I	287	136	151	
II	52	13	39	**<0.001**
III	111	65	46	
IV	55	39	16	
Lymph node metastasis				
Yes	227	84	143	
No	229	152	77	**<0.001**
Unknown	49	17	32	
Distant metastases				
Yes	280	157	123	
No	9	3	6	**0.009**
Unknown	216	93	123	
Location				
Left lobe	176	89	87	
Right lobe	219	105	114	0.348
Bilateral	88	44	44	
Isthmus	22	15	7	
Progression state				
Yes	53	35	18	**0.014**
No	452	218	234	

*P-values less than 0.05 are bold*.

**Table 4:** Univariate and Multivariate Cox logistic regression analysis of FN1 for predicting PFS in TCGA cohort (PFS, progression free survival; TCGA, The Cancer Genome Atlas).

**Variable**	**Univariate**			**Multivariate**		
	**HR**	**(95% CI)**	***P***	**HR**	**(95% CI)**	***P***
Age (ref. <60)	2.10	1.20~3.70	**0.007**	1.49	0.78~2.80	0.225
Gender (ref. Male)	0.58	0.33~1.00	0.055	0.69	0.39~1.20	0.211
Clinical stage (ref. I–II)	2.60	1.50~4.40	**<0.001**	1.94	1.03~3.60	**0.039**
Lymph node metastasis (ref. No)	1.60	0.91~2.80	0.11	1.13	0.62~2.10	0.687
Distant metastases (ref. No)	1.50	0.88~2.60	0.13	1.65	0.95~2.90	0.076
Location (ref. left and right lode)	0.90	0.45~1.80	0.77	0.84	0.42~1.70	0.632
FN1 expression (ref. low)	1.90	1.10~3.40	**0.022**	1.83	1.01~3.30	**0.046**

*P-values less than 0.05 are bold*.

### Analysis of the Potential Genetic and Epigenetic Alterations Underlying *FN1* Dysregulation

**Figure 4:**
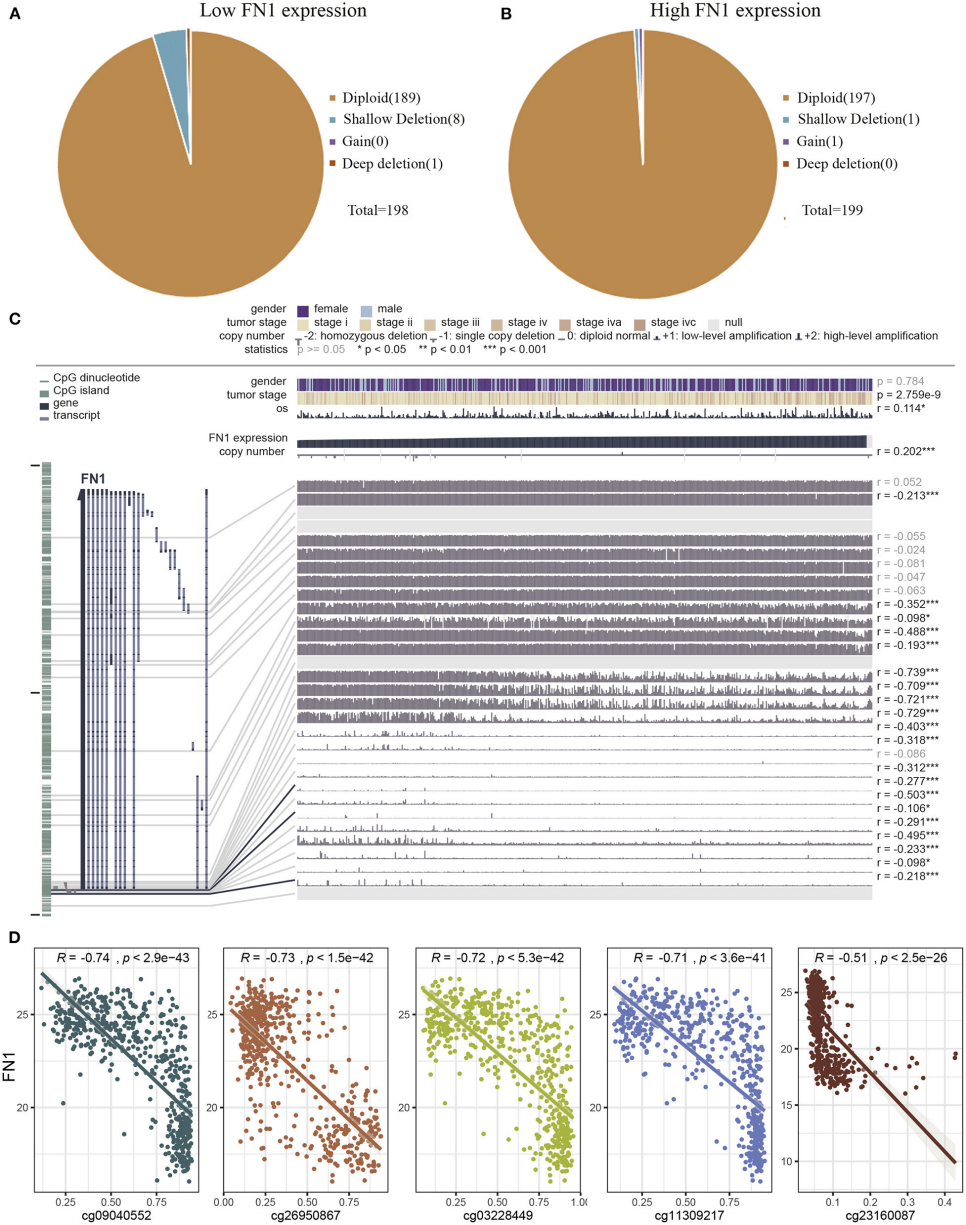
Analysis of the potential genetic and epigenetic alterations associated with FN1 dysregulation. **(A,B)** The CNAs in FN1-high and low expression groups. **(C)** Analysis of CpG island methylation and abnormal FN1 expression using TCGA-THCA dataset. **(D)** Correlation between FN1 expression and CpG island methylation was performed. *p < 0.05, **p < 0.01, ***p < 0.001.

Next, we investigated the underlying mechanism of *FN1* dysregulation in THCA. To determine whether copy number alterations (CNAs) are responsible for the abnormal expression of *FN1* in THCA, we analyzed 397 cases from the TCGA database for which CNAs data was available. No differences were observed for CNAs in the FN1-high and FN1-low groups (Figures 4A,B). Another mechanism that could underlie the altered *FN1* expression profile observed in THCA samples is promoter hypermethylation, which plays an important role in the occurrence and development of several types of tumors (34, 35). To investigate whether DNA methylation results in FN1 dysfunction, we examined the status of CpG sites in 507 THCA cases from the TCGA database using the tool MEXPRESS. We found that 27 CpG sites had associated data; among which 20 CpG sites showed a negative correlation with *FN1* expression (Figure 4C). The Pearson correlation coefficient was calculated for the five CpG sites with the highest correlation coefficient, including cg21494132, cg09040552, cg11309217, cg03228449, and cg03228449 (Figure 4D, all *P* < 0.05). Our results demonstrate the correlation between DNA methylation and abnormal expression levels of *FN1* in THCA, highlighting the need for further investigation of the underlying mechanism.

### Correlation Between FN1 and Inflammatory Activities

**Figure 5:**
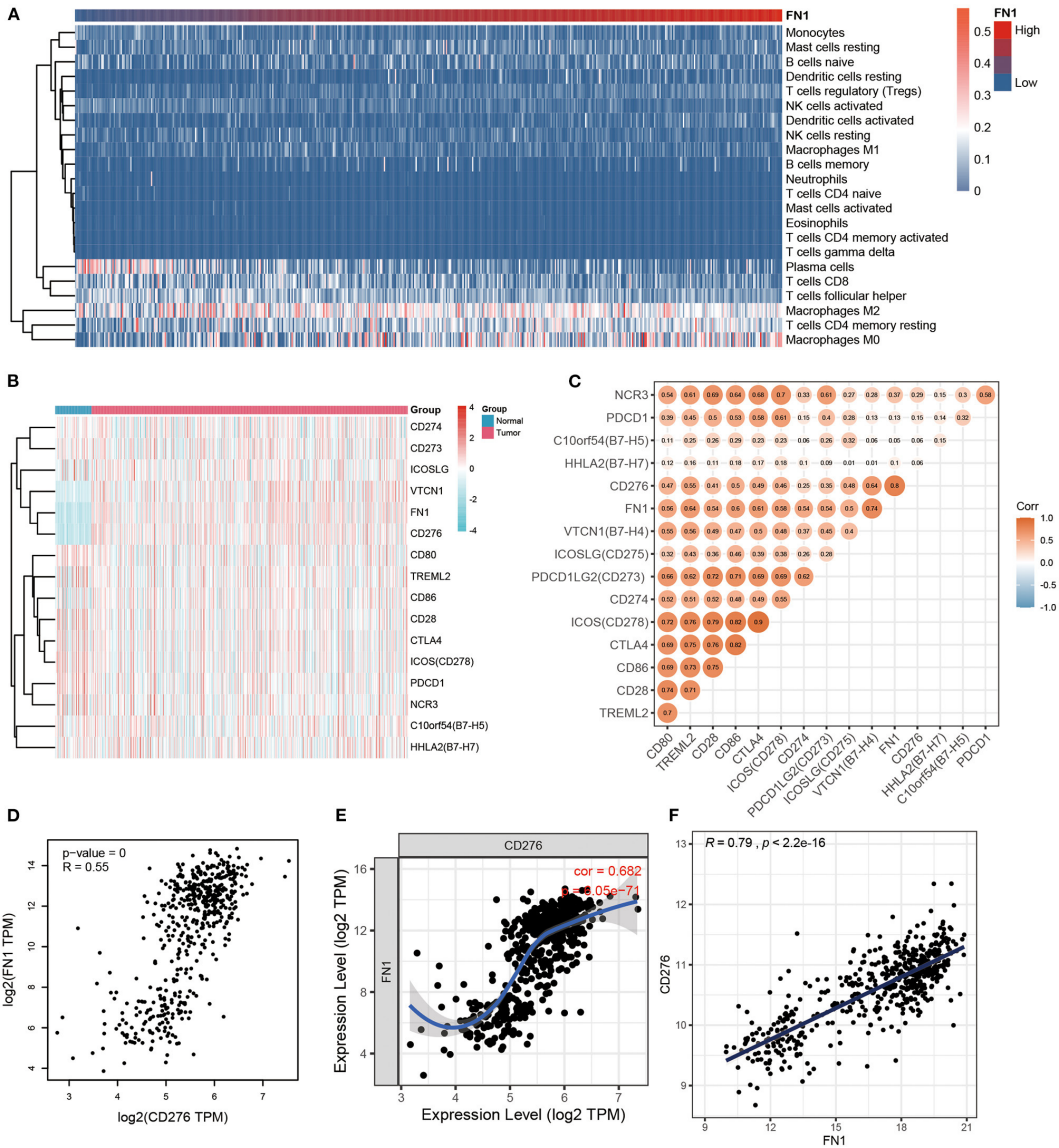
The correlation between FN1 and inflammatory activities. **(A)** The heatmap of immune infiltration analysis. **(B)** The coexpression between FN1 and the B7-CD28 ligand-receptor family. **(C)** The correlation between FN1, CD273, CD274, CD275, CD276, and B7-H4 in THCA cohort. The correlation between FN1 and CD276 analyzed by THCA datasets **(D)**, TIMER **(E)**, and GEPIA **(F)**.

Considering the strong association between *FN1* expression levels and THCA prognosis, we hypothesized that FN1 may be associated with inflammatory responses, leading to enhanced survival rates. To identify the FN1 associated immune signature in THCA, we assessed the degree of immune infiltration with CIBERSORT. *FN1* expression was closely correlated to the degree of M2 macrophages, resting memory CD4^+^ T cells, follicular helper T cells, and CD8^+^ T cell infiltration. The increase in *FN1* expression was associated with an increase of the proportion of M2 macrophages and resting memory CD4^+^ T cells and a decrease of the proportion of follicular helper T cells and CD8^+^ T cells (Figure 5A). To further investigate the correlation between *FN1* and inflammation, we analyzed the co-expression of *FN1* and members of the B7-CD28 ligand-receptor family, including *CD274, CD80, CD86, CD276, CD273, CD275, B7-H4, B7-H5, CD28, B7-H7, CD152, CD279, CD278, TLT-2*, and *NKp30*, which are closely correlated to T cell function. The result demonstrated that FN1 exhibited a significant co-expression trend with CD273, CD274, CD275, CD276 and B7-H4 (Figure 5B). Furthermore, we investigated the correlation between *FN1, CD273, CD274, CD275, CD276*, and *B7-H4* expression in the THCA cohort, and found a close positive correlation between *FN1* and *CD276* expression (*R* = 0.55, *P* = 0) (Figures 5C,D). This result was validated using TIMER and GEPIA, which provided *R*-values of 0.682 and 0.79, respectively, for the correlation between *FN1* and *CD276* in THCA (*P* < 0.001) (Figures 5E,F).

### Down-Regulation of *FN1* Inhibited Cell Proliferation and Invasion and Decreased *CD276* Expression Levels in THCA Samples

**Figure 6:**
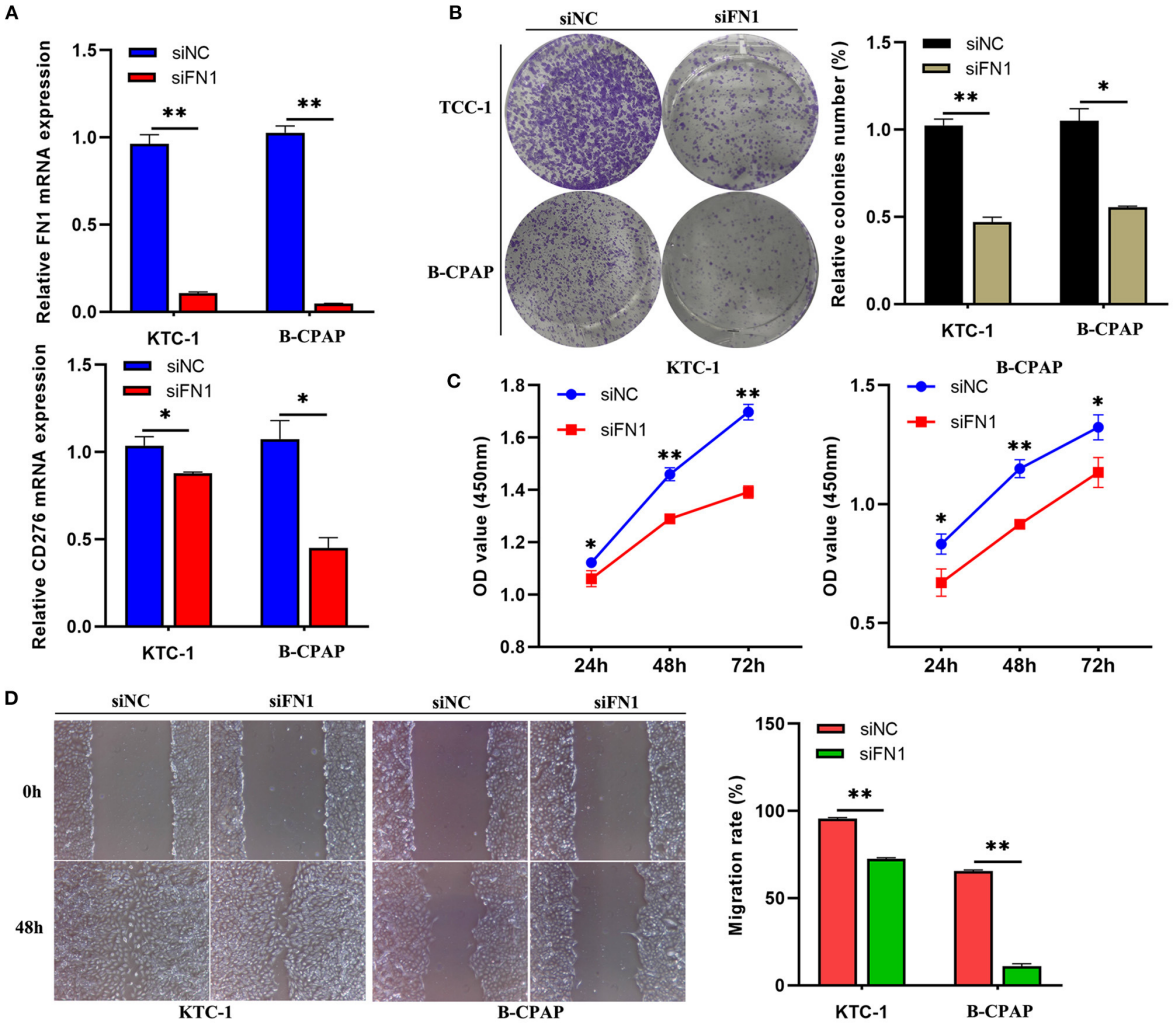
Down-regulation of FN1 suppressed THCA cell migration and invasion *in vitro*. **(A)** qRT-PCR showing the efficient depletion of FN1 expression and the expression of CD276 in B-CPAP and KTC-1 cells compared with siNC transfection. Representative photo-images (left) and histograms (right) of the effect of siFN1 on the clone formation **(B)** and migration **(D)** of B-CPAP and KTC-1 cells. **(C)** The proliferative ability of B-CPAP and KTC-1 cells after transfection was evaluated by CCK-8 assay. *p < 0.05, **p < 0.001.

To explore the biological significance of *FN1* in THCA tumorigenesis, KTC-1 and B-CPAP cells were transfected with siRNA targeting *FN1* (siFN1) or negative control siRNA (siNC). Efficient depletion of *FN1* expression was confirmed *via* RT-qPCR (*P* < 0.05, Figure 6A). Moreover, we found that downregulation of *FN1* significantly reduced the expression of *CD276* in KTC-1 and B-CPAP cells compared with siNC transfection (Figure 6A), indicating that that the interactions between FN1 and CD276 could be a potential mechanism for the correlation of FN1 expression with immune infiltration and poor prognosis in THCA. Then, we studied the effect of *FN1* on THCA cell proliferation and invasion *in vitro*. The wound-healing assay, cell viability, and cytotoxicity CCK-8 assay, and clone formation assay revealed that downregulation of *FN1* in both cell types significantly inhibited cell proliferation and invasion compared to that in the control cells (*P* < 0.05, Figures 6B–D). These results suggest that the downregulation of *FN1* reduces the viability of THCA cell lines.

### FN1 Is Positively Correlated With CD276

**Figure 7:**
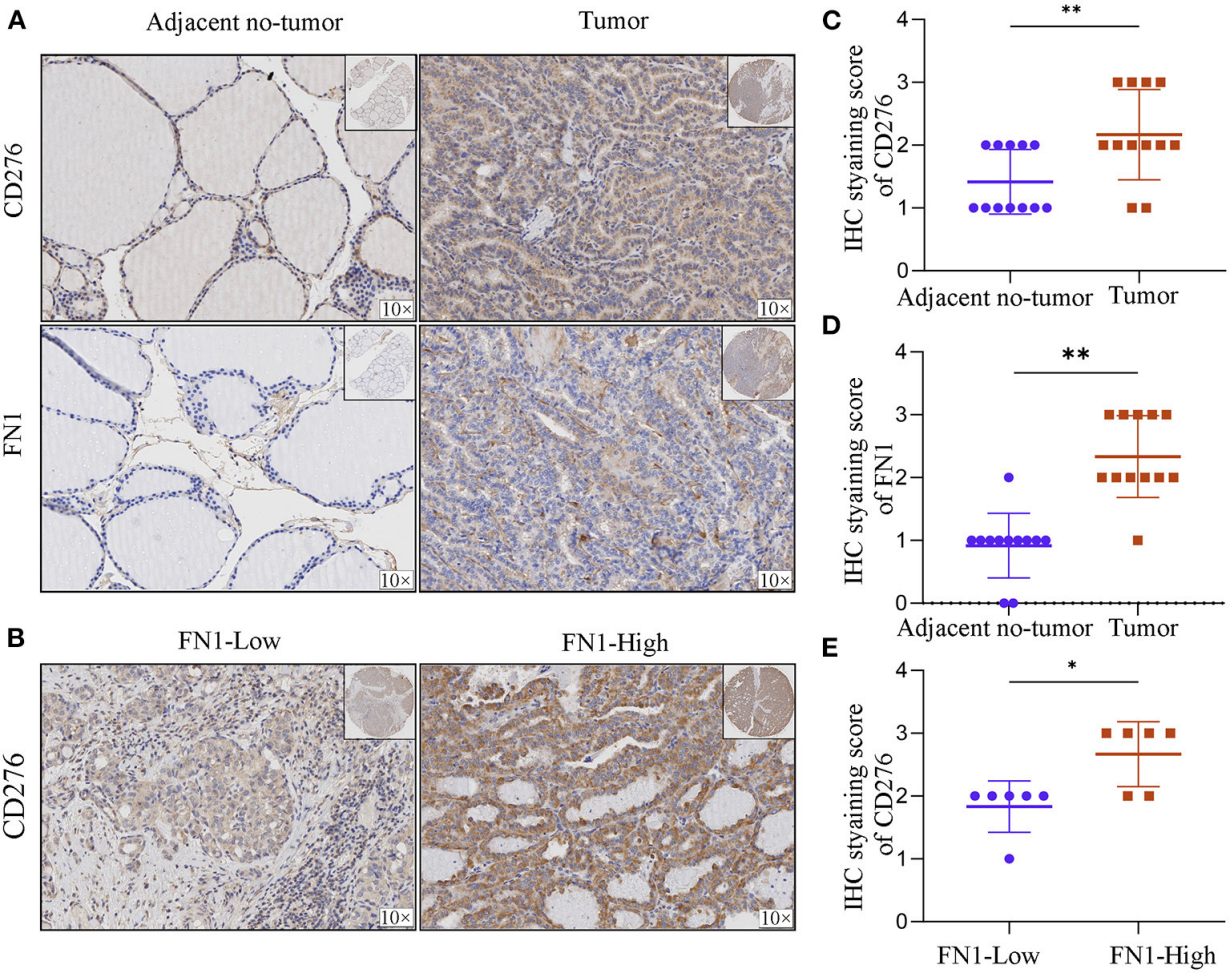
The correlation between FN1 and CD276 in THCA. **(A)** Representative IHC staining of CD276 and FN1 in tumors and adjacent no-tumor tissues from THCA patients. **(B)** Representative IHC staining of CD276 in tumor tissues from FN1-high and low patient groups. IHC score of CD276 **(C)** and FN1 **(D)** in adjacent no-tumor tissues and tumor tissues form patients with THCA. **(E)** IHC score of CD276 in tumor tissues from FN1-high and low patient groups, *p < 0.05, **P < 0.01.

In order to assess FN1 correlation with CD276, we analyzed FN1 and CD276 expression in tumor sites and the adjacent no-tumor samples (Figure 7A). We found that FN1 and CD276 showed significantly higher expression in tumor sites than in the adjacent no-tumor samples (*P* < 0.05, Figures 7C,D). Furthermore, we divided the tumor sites into two groups according to FN1 expression and found that CD276 levels were positively correlated with the level of FN1(*P* < 0.05, Figures 7B,E).

## Discussion

*FN1* is a member of the FN family widely expressed in multiple cell types and is involved in cellular adhesion and migration processes (36). Here, we report that variations in *FN1* expression levels correlate with prognosis in THCA patients. High expression levels of *FN1* are associated with a poorer prognosis in THCA, indicating that *FN1* expression could be used to predict tumor patient' prognosis. Furthermore, our analysis show that immune infiltration levels and immune markers are correlated with *FN1* expression level in THCA, suggesting a potential role of *FN1* in tumor immunology and its possible used as a cancer biomarker.

In this study, we screened DEGs from three GEO datasets based on functional enrichment analysis and PPI network maps. *FN1*, which is closely correlated to disease-free survival, was identified as a hub gene. In addition, we found that the promoter region of *FN1* had significantly lower methylation levels (*P* < 0.05) in THCA than in normal thyroid tissues. GSEA analysis also showed that *FN1* plays a role in the chemokine signaling pathway, cytokine receptor interaction, natural killer cell-mediated cytotoxicity, and T cell receptor signaling pathway, which is closely correlated to tumorigenesis. Importantly, immune infiltration analysis showed that immune infiltration level and diverse immune marker sets were correlated with *FN1* expression level. Therefore, *FN1* can be a potential immunity-related biomarker and therapeutic target in THCA. Moreover, using quantitative proteomic approaches, previous studies have proved that *FN1* can be a potential novel candidate prognostic biomarker in THCA (37). However, these studies did not specify the exact range of effects of genes on disease prognosis, lacked certain clinical significance. In this study, three GEO datasets were combined to screen the hub genes, providing results with a high statistical and clinical significance. Our research further demonstrates that *FN1* is a potential prognostic biomarker and therapeutic target in THCA from the perspective of DNA methylation and tumor immunology.

The important aspect of this study is that *FN1* expression is correlated with diverse immune infiltration level in THCA. An increase in *FN1* expression level was positively correlated with the proportion of M2 macrophages and resting memory CD4^+^ T cells but negatively correlated with the proportion of follicular helper T cells and CD8^+^ T cells. To further investigate the correlation between *FN1* and inflammatory activities, the correlation between *FN1* and members of the B7-CD28 ligand-receptor family was analyzed, and a close positive correlation between *FN1* and *CD276* was identified. *CD276*, a member of the B7 superfamily, has been previously identified as a poor prognostic factor. A previous study demonstrated that *CD276*, expressed in multiple tumor lines, tumor-infiltrating dendritic cells, and macrophages, can inhibit T-cell activation and autoimmunity (38). Therefore, the interactions between *FN1* and *CD276* could be a potential mechanism underlying the correlation of *FN1* expression with immune infiltration and poor prognosis in THCA.

In summary, increased *FN1* expression correlated with poor prognosis and altered immune infiltration levels in THCA, indicating that *FN1* is a potential immunity-related biomarker. This study was based on a statistical analysis of bioinformatics methods, and the conclusions obtained were supported by experimental data and multiple databases. Therefore, it is reasonable to believe that FN1 plays an important role in the diagnosis, treatment, and prognosis of THCA.

## Conclusion

In summary, we performed a comprehensive analysis using the TCGA dataset and multiple online databases and identified *FN1* as a potential immunity-related biomarker and a prognostic marker in THCA. Our results suggest FN1 has a significant part in the diagnosis, treatment, and prognosis of THCA, highlighting the necessity of future clinical research in the topic.

## Data Availability Statement

The datasets presented in this study can be found in online repositories. The names of the repository/repositories and accession number(s) can be found in the article/Supplementary Material.

## Ethics Statement

All the patients' data involved in this study is open source which is freely available in the public research databases including the Cancer Genome Atlas (TCGA) and Gene Expression Omnibus (GEO). The application of the public data is already properly anonymized and informed consent was also obtained at the time of the original data collection. Samples from cancer patients were obtained from thyroid cancer arrays (DC-Thy11004 Avira Biotechnology Co., Ltd., China). The data is already properly anonymized and informed consent was also obtained at the time of the original data collection.

## Author Contributions

Q-SG is responsible for the acquisition of the data, analysis and interpretation of the data. TH and L-FL are responsible for the drafting of the manuscript, and statistical analysis. Z-BS, W-HX,and JZ contributed to the critical revision of the manuscript. All authors contributed to the article and approved the submitted version.

## Funding

This work was supported by the Collaborative Innovation Major Project of Zhengzhou (Grant No. 20XTZX08017), National Natural Science Foundation of China (Grant Nos. 82002433 and 82002998), Science and Technology Project of Henan Provincial Department of Education (Grant Nos. 18A320044 and 21A320036), Henan Province Medical Science and Technology Research Project Joint Construction Project (Grant Nos. LHGJ20190003, LHGJ20190055, and LHGJ20190042), First-class Postdoctoral Research Grant in Henan Province (Grant No. 201901007), and Youth Talent Support Project of Henan Province (Grant No. 2021HYTP045) also support this work.

## Conflict of Interest

The authors declare that the research was conducted in the absence of any commercial or financial relationships that could be construed as a potential conflict of interest.

## Publisher's Note

All claims expressed in this article are solely those of the authors and do not necessarily represent those of their affiliated organizations, or those of the publisher, the editors and the reviewers. Any product that may be evaluated in this article, or claim that may be made by its manufacturer, is not guaranteed or endorsed by the publisher.

## Supplementary Material

Supplementary Figure 1 Flow diagram of the study.

Supplementary Table 1 The significantly enriched GO terms of the common DEGs (BP, biological process; CC, cellular component; DEG, differentially expressed gene; GO, Gene Ontology; MF, molecular function).

## Abbreviations

THCA: Thyroid cancer
GEO: the Gene Expression Omnibus database
DEGs: the differentially expressed genes
DAVID: the Database for Annotation, Visualization and Integrated Discovery
STRING: the Retrieval of Interacting Genes
PPI: the protein–protein interaction
GEPIA: Gene expression profiling and interactive analyses
PTC: papillary thyroid carcinoma
FTC: follicular thyroid carcinoma
ATC: undifferentiated thyroid carcinoma
MTC: medullary thyroid carcinoma
IHC: Immunohistochemistry.

## References

[1] Bonhomme B Godbert Y Perot G Al GA Bardet S Belleannee G. Molecular pathology of anaplastic thyroid carcinomas: a retrospective study of 144 cases. Thyroid. (2017) 27:682–92. 10.1089/thy.2016.0254 28351340

[2] Davies L Welch HG. Current thyroid cancer trends in the United States. JAMA Otolaryngol. (2014) 140:317–22. 10.1001/jamaoto.2014.1 24557566

[3] Zhao L Pang P Zang L Luo Y Wang F Yang G. Features and trends of thyroid cancer in patients with thyroidectomies in Beijing, China between 1994 and 2015: a retrospective study. BMJ Open. (2019) 9:e0233341. 10.1136/bmjopen-2018-023334 30782703 PMC6347868

[4] Aschebrook-Kilfoy B Ward MH Sabra MM Devesa SS. Thyroid cancer incidence patterns in the United States by histologic type, 1992-2006. Thyroid. (2011) 21:125–34. 10.1089/thy.2010.0021 21186939 PMC3025182

[5] Chen AY Bernet VJ Carty SE Davies TF Ganly I Inabnet WR. American Thyroid Association statement on optimal surgical management of goiter. Thyroid. (2014) 24:181–9. 10.1089/thy.2013.0291 24295043

[6] Wang C. Sun P. Yang L. i. J. Yang W. Feng J. (2016). Z, et al. Strategies of laparoscopic thyroidectomy for treatment of substernal goiter via areola approach. Surg Endosc. 30, 4721–4730. 10.1007/s00464-016-4814-0 27005286

[7] Sipos JA Mazzaferri EL. Thyroid cancer epidemiology and prognostic variables. Clin Oncol. (2010) 22:395–404. 10.1016/j.clon.2010.05.004 20627675

[8] Naoum GE Morkos M Kim B Arafat W. Novel targeted therapies and immunotherapy for advanced thyroid cancers. Mol Cancer. (2018) 17:51. 10.1186/s12943-018-0786-0 29455653 PMC5817719

[9] Mlecnik B Van den Eynde M Bindea G Church SE Vasaturo A Fredriksen T. Comprehensive intrametastatic immune quantification and major impact of immunoscore on survival. J Natl Cancer Inst. (2018) 110:djx123. 10.1093/jnci/djx123 28922789

[10] Pankov R Yamada KM. Fibronectin at a glance. J Cell Sci. (2002) 115(Pt 20):3861–3. 10.1242/jcs.00059 12244123

[11] Chen Z Tao Q Qiao B Zhang L. Silencing of LINC01116 suppresses the development of oral squamous cell carcinoma by up-regulating microRNA-136 to inhibit FN1. Cancer Manag Res. (2019) 11:6043–59. 10.2147/CMAR.S197583 31308744 PMC6613355

[12] Waalkes S Atschekzei F Kramer MW Hennenlotter J Vetter G Becker JU. Fibronectin 1 mRNA expression correlates with advanced disease in renal cancer. BMC Cancer. (2010) 10:503. 10.1186/1471-2407-10-503 20860816 PMC2949811

[13] Sponziello M Rosignolo F Celano M Maggisano V Pecce V Rose D. RF, et al. Fibronectin-1 expression is increased in aggressive thyroid cancer and favors the migration and invasion of cancer cells. Mol Cell Endocrinol. (2016) 431:123–32. 10.1016/j.mce.2016.05.007 27173027

[14] Glasner A Levi A Enk J Isaacson B Viukov S Orlanski S. NKp46 receptor-mediated interferon-γ production by natural killer cells increases fibronectin 1 to alter tumor architecture and control metastasis. Immunity. (2018) 48:107–19. 10.1016/j.immuni.2017.12.007 29329948

[15] Zhang DL Wang JM Wu T Du X Yan J. Du ZX, et al. BAG5 promotes invasion of papillary thyroid cancer cells via upregulation of fibronectin 1 at the translational level. Biochim Biophys Acta Mol Cell Res. (2020) 1867:118715. 10.1016/j.bbamcr.2020.118715 32275930

[16] Yu S Yu X Sun L Zheng Y Chen L Xu H. GBP2 enhances glioblastoma invasion through Stat3/fibronectin pathway. Oncogene. (2020) 39:5042–55. 10.1038/s41388-020-1348-7 32518375

[17] Rago T Scutari M Loiacono V Santini F Tonacchera M Torregrossa L. Low elasticity of thyroid nodules on ultrasound elastography is correlated with malignancy, degree of fibrosis, and high expression of galectin-3 and fibronectin-1. Thyroid. (2017) 27:103–10. 10.1089/thy.2016.0341 27809694

[18] Goldman MJ Craft B Hastie M Repečka K McDade F Kamath A. Visualizing and interpreting cancer genomics data via the Xena platform. Nat Biotechnol. (2020) 38:675–8. 10.1038/s41587-020-0546-8 32444850 PMC7386072

[19] Tomas G Tarabichi M Gacquer D Hebrant A Dom G Dumont JE. A general method to derive robust organ-specific gene expression-based differentiation indices: application to thyroid cancer diagnostic. Oncogene. (2012) 31:4490–8. 10.1038/onc.2011.626 22266856

[20] Rusinek D Swierniak M Chmielik E Kowal M Kowalska M Cyplinska R. BRAFV600E-associated gene expression profile: early changes in the transcriptome, based on a transgenic mouse model of papillary thyroid carcinoma. PLoS ONE. (2015) 10:e143688. 10.1371/journal.pone.0143688 26625260 PMC4666467

[21] Tarabichi M Saiselet M Tresallet C Hoang C Larsimont D Andry G. Revisiting the transcriptional analysis of primary tumours and associated nodal metastases with enhanced biological and statistical controls: application to thyroid cancer. Br J Cancer. (2015) 112:1665–74. 10.1038/bjc.2014.665 25965298 PMC4430711

[22] Kameshwar AK Qin W. Metadata analysis of phanerochaete chrysosporium gene expression data identified common CAZymes encoding gene expression profiles involved in cellulose and hemicellulose degradation. Int J Biol Sci. (2017) 13:85–99. 10.7150/ijbs.17390 28123349 PMC5264264

[23] Khan A Mathelier A. Intervene: a tool for intersection and visualization of multiple gene or genomic region sets. BMC Bioinformatics. (2017) 18:287. 10.1186/s12859-017-1708-7 28569135 PMC5452382

[24] Chen L Zhang YH Lu G Huang T Cai YD. Analysis of cancer-related lncRNAs using gene ontology and KEGG pathways. Artif Intell Med. (2017) 76:27–36. 10.1016/j.artmed.2017.02.001 28363286

[25] Zhao Z Bai J Wu A Wang Y Zhang J Wang Z. Co-LncRNA: investigating the lncRNA combinatorial effects in GO annotations and KEGG pathways based on human RNA-Seq data. Database. (2015) 2015:bav082. 10.1093/database/bav082 26363020 PMC4565967

[26] Cook HV Doncheva NT Szklarczyk D von Mering C Jensen LJ. Viruses.STRING: a virus-host protein-protein interaction database. Viruses. (2018) 10:519. 10.3390/v10100519 30249048 PMC6213343

[27] Doncheva NT Morris JH Gorodkin J Jensen LJ. Cytoscape StringApp: network analysis and visualization of proteomics data. J Proteome Res. (2019) 18:623–32. 10.1021/acs.jproteome.8b00702 30450911 PMC6800166

[28] Rhodes DR Yu J Shanker K Deshpande N Varambally R Ghosh D. ONCOMINE: a cancer microarray database and integrated data-mining platform. Neoplasia. (2004) 6:1–6. 10.1016/S1476-5586(04)80047-2 15068665 PMC1635162

[29] Chandrashekar DS Bashel B Balasubramanya S Creighton CJ Ponce-Rodriguez I Chakravarthi B. UALCAN: a portal for facilitating tumor subgroup gene expression and survival analyses. Neoplasia. (2017) 19:649–58. 10.1016/j.neo.2017.05.002 28732212 PMC5516091

[30] Koch A Jeschke J Van Criekinge W van Engeland M Meyer De T. MEXPRESS update 2019. Nucleic Acids Res. (2019) 47:W561–5. 10.1093/nar/gkz445 31114869 PMC6602516

[31] Huang WY Hsu SD Huang HY Sun YM Chou CH Weng SL. A database of DNA methylation and gene expression in human cancer. Nucleic Acids Res. (2015) 43:D856–61. 10.1093/nar/gku1151 25398901 PMC4383953

[32] Newman AM Steen CB Liu CL Gentles AJ Chaudhuri AA Scherer F. Determining cell type abundance and expression from bulk tissues with digital cytometry. Nat Biotechnol. (2019) 37:773–82. 10.1038/s41587-019-0114-2 31061481 PMC6610714

[33] Tang Z Li C Kang B Gao G Li C Zhang Z. GEPIA: a web server for cancer and normal gene expression profiling and interactive analyses. Nucleic Acids Res. (2017) 45:W98–102. 10.1093/nar/gkx247 28407145 PMC5570223

[34] Morgan AE Davies TJ Mc AM. The role of DNA methylation in ageing and cancer. Proc Nutr Soc. (2018) 77:412–22. 10.1017/S0029665118000150 29708096

[35] Flanagan JM Wilson A Koo C Masrour N Gallon J Loomis E. Platinum-based chemotherapy induces methylation changes in blood DNA associated with overall survival in patients with ovarian cancer. Clin Cancer Res. (2017) 23:2213–22. 10.1158/1078-0432.CCR-16-1754 27663594

[36] Ruoslahti E. Fibronectin in cell adhesion and invasion. Cancer Metastasis Rev. (1984) 3:43–51. 10.1007/BF00047692 6324988

[37] Zhan S Li J Wang T Ge W. Quantitative proteomics analysis of sporadic medullary thyroid cancer reveals FN1 as a potential novel candidate prognostic biomarker. Oncologist. (2018) 23:1415–25. 10.1634/theoncologist.2017-0399 29739896 PMC6292558

[38] Lee YH Martin-Orozco N Zheng P Li J Zhang P Tan H. Inhibition of the B7-H3 immune checkpoint limits tumor growth by enhancing cytotoxic lymphocyte function. Cell Res. (2017) 27:1034–45. 10.1038/cr.2017.90 28685773 PMC5539354

[39] Li T Fan J Wang B Traugh N Chen Q Liu JS. TIMER: a web server for comprehensive analysis of tumor-infiltrating immune cells. Cancer Res. (2017) 77:e108–10. 10.1158/0008-5472.CAN-17-0307 29092952 PMC6042652

