# Supplementary figures and images for: Over-Expression and Prognostic Significance of FN1, Correlating With Immune Infiltrates in Thyroid Cancer
Source: Front Med (Lausanne). 2022 Jan 24;8:812278. doi: 10.3389/fmed.2021.812278 (PMC8818687; doi:10.3389/fmed.2021.812278)

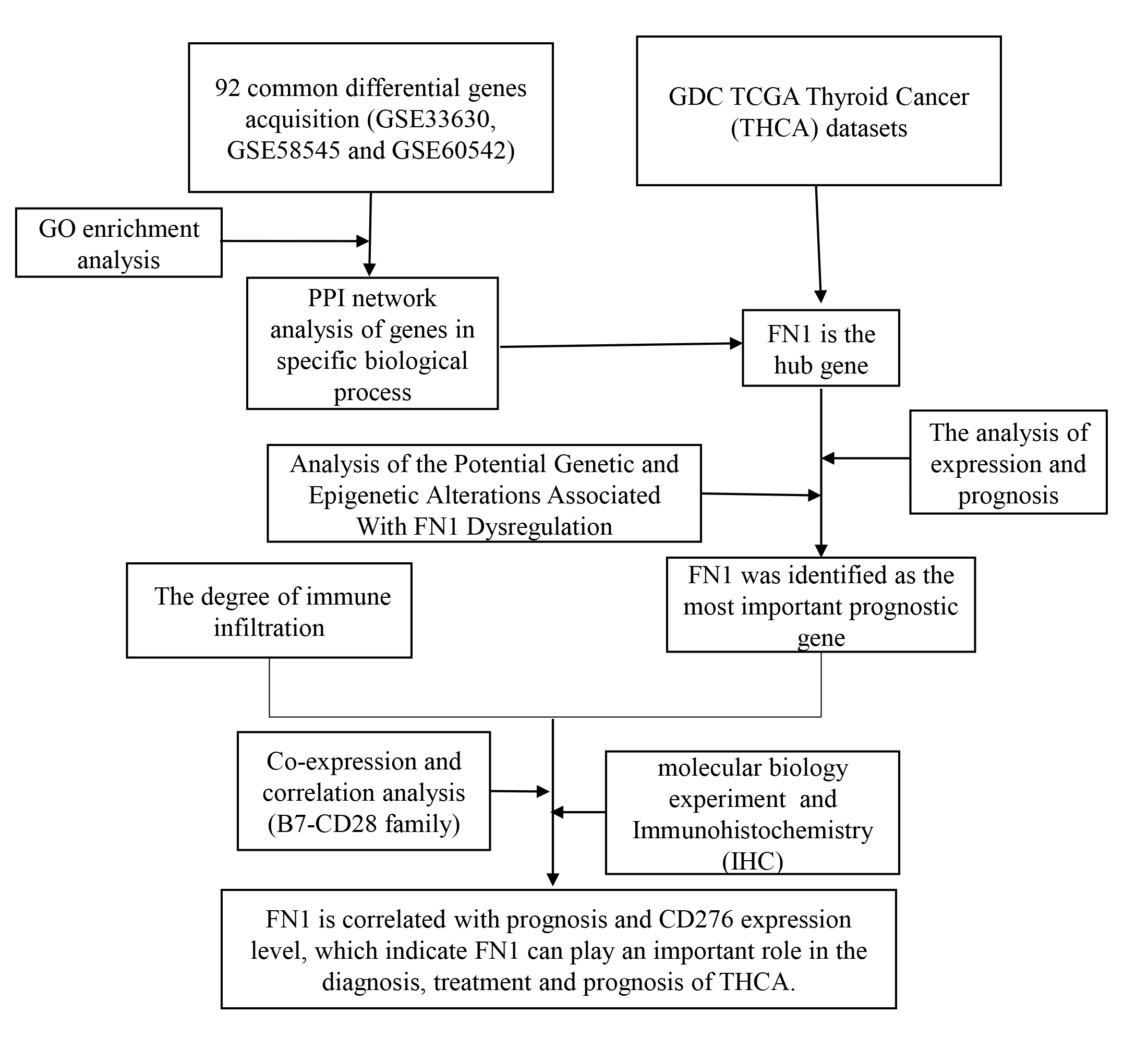

Supplement: Supplementary Figure 1 — Flow diagram of the study. [file Image_1.TIF]
