# Supplementary material for: Over-Expression and Prognostic Significance of FN1, Correlating With Immune Infiltrates in Thyroid Cancer
Source: Front Med (Lausanne). 2022 Jan 24;8:812278. doi: 10.3389/fmed.2021.812278 (PMC8818687; doi:10.3389/fmed.2021.812278)
Supplement: Supplementary Table 1 — The significantly enriched GO terms of the common DEGs (BP, biological process; CC, cellular component; DEG, differentially expressed gene; GO, Gene Ontology; MF, molecular function). [file Table_1.DOCX]

**Table S1** The significantly enriched GO terms of the common DEGs (BP, biological process; CC, cellular component; DEG, differentially expressed gene; GO, Gene Ontology; MF, molecular function)

| **Category** | **Term** | **Count** | **P Value** |
| --- | --- | --- | --- |
| GOTERM_MF_DIRECT | GO:0042803~protein  homodimerization activity | 11 | 5.87E-03 |
| GOTERM_MF_DIRECT | GO:0042802~identical protein  binding | 9 | 4.88E-02 |
| GOTERM_MF_DIRECT | GO:0005509~calcium ion binding | 8 | 9.20E-02 |
| GOTERM_MF_DIRECT | GO:0002020~protease binding | 7 | 1.75E-05 |
| GOTERM_MF_DIRECT | GO:0008201~heparin binding | 5 | 1.10E-02 |
| GOTERM_CC_DIRECT | GO:0005886~plasma membrane | 38 | 1.90E-04 |
| GOTERM_CC_DIRECT | GO:0070062~extracellular exosome | 30 | 1.24E-04 |
| GOTERM_CC_DIRECT | GO:0005576~extracellular region | 25 | 1.45E-06 |
| GOTERM_CC_DIRECT | GO:0005615~extracellular space | 24 | 2.39E-07 |
| GOTERM_CC_DIRECT | GO:0005887~integral component of  plasma membrane | 18 | 8.39E-04 |
| GOTERM_CC_DIRECT | GO:0005578~proteinaceous  extracellular matrix | 10 | 1.01E-05 |
| GOTERM_CC_DIRECT | GO:0043025~neuronal cell body | 10 | 3.61E-05 |
| GOTERM_CC_DIRECT | GO:0009986~cell surface | 8 | 2.20E-02 |
| GOTERM_CC_DIRECT | GO:0016324~apical plasma  membrane | 7 | 3.96E-03 |
| GOTERM_CC_DIRECT | GO:0031093~platelet alpha granule  lumen | 5 | 1.85E-04 |
| GOTERM_CC_DIRECT | GO:0030018~Z disc | 5 | 3.25E-03 |
| GOTERM_BP_DIRECT | GO:0007155~cell adhesion | 14 | 1.61E-06 |
| GOTERM_BP_DIRECT | GO:0007165~signal transduction | 14 | 1.25E-02 |
| GOTERM_BP_DIRECT | GO:0030198~extracellular matrix  organization | 7 | 8.14E-04 |
| GOTERM_BP_DIRECT | GO:0010628~positive regulation of  gene expression | 7 | 3.53E-03 |
| GOTERM_BP_DIRECT | GO:0008284~positive regulation of  cell proliferation | 7 | 4.67E-02 |
| GOTERM_BP_DIRECT | GO:0007596~blood coagulation | 6 | 3.74E-03 |
| GOTERM_BP_DIRECT | GO:0042060~wound healing | 5 | 1.05E-03 |
| GOTERM_BP_DIRECT | GO:0002576~platelet degranulation | 5 | 2.67E-03 |
